# Study Protocol—Coping With the Pandemics: What Works Best to Reduce Anxiety and Depressive Symptoms

**DOI:** 10.3389/fpsyt.2021.642763

**Published:** 2021-07-02

**Authors:** Lydia Fortea, Aleix Solanes, Edith Pomarol-Clotet, Maria Angeles Garcia-Leon, Adriana Fortea, Carla Torrent, Cristina Varo, C. Mar Bonnin, Laura Montejo, Jordi Alonso, Susana Carmona, Pau Soldevila-Matías, Irene Alustiza, Daniel Arbós, Diego Hidalgo-Mazzei, Iria Grande, Eduard Vieta, Miquel Àngel Fullana, Joaquim Radua

**Affiliations:** ^1^Imaging of Mood- and Anxiety-Related Disorders (IMARD) Group, Institut d'Investigacions Biomèdiques August Pi i Sunyer (IDIBAPS), Barcelona, Spain; ^2^Centro de Investigación Biomédica en Red de Salud Mental (CIBERSAM), Instituto de Salud Carlos III, Madrid, Spain; ^3^Department of Medicine, University of Barcelona, Barcelona, Spain; ^4^Department of Psychiatry and Forensic Medicine, Autonomous University of Barcelona, Barcelona, Spain; ^5^FIDMAG Research Foundation, Barcelona, Spain; ^6^Multimodal Neuroimaging in High Risk and Early Psychosis, Institut d'Investigacions Biomèdiques August Pi i Sunyer (IDIBAPS), Barcelona, Spain; ^7^Department of Child and Adolescent Psychiatry, Institute of Neurosciences, Hospital Clínic de Barcelona, Barcelona, Spain; ^8^Adult Psychiatry and Psychology Department, Institute of Neurosciences, Hospital Clínic de Barcelona, Barcelona, Spain; ^9^Bipolar and Depressive Disorders, Institut d'Investigacions Biomèdiques August Pi i Sunyer (IDIBAPS), Barcelona, Spain; ^10^Health Services Research Unit, Institut Hospital del Mar d'Investigacions Mèdiques (IMIM), Barcelona, Spain; ^11^Department of Experimental and Health Sciences, Universitat Pompeu Fabra (UPF), Barcelona, Spain; ^12^Centro de Investigación Biomédica en Red de Epidemiología y Salud Pública (CIBERESP), Instituto de Salud Carlos III, Madrid, Spain; ^13^Instituto de Investigación Sanitaria Gregorio Marañón, Madrid, Spain; ^14^Departamento de Bioingeniería e Ingeniería Aeroespacial, Universidad Carlos III de Madrid, Getafe, Spain; ^15^Department of Basic Psychology, Faculty of Psychology, University of Valencia, Valencia, Spain; ^16^Department of Psychiatry and Clinical Psychology, Clínica Universidad de Navarra, Pamplona, Spain; ^17^Instituto de Investigación Sanitaria de Navarra (IDISNA), Navarra, Spain; ^18^Communication Office, Institut d'Investigacions Biomèdiques August Pi i Sunyer (IDIBAPS), Barcelona, Spain; ^19^Early Psychosis: Interventions and Clinical-Detection (EPIC) Lab, Department of Psychosis Studies, Institute of Psychiatry, Psychology & Neuroscience, King's College London, London, United Kingdom; ^20^Centre for Psychiatric Research and Education, Department of Clinical Neuroscience, Karolinska Institutet, Stockholm, Sweden

**Keywords:** COVID-19, anxiety, depressive symptoms, coping behaviors, longitudinal study

## Abstract

**Background:** The coronavirus disease 2019 (COVID-19) pandemic and lockdown might increase anxiety and depressive symptoms in most individuals. Health bodies recommend several coping behaviors to protect against such symptoms, but evidence on the relationship between these behaviors and symptoms mostly comes from cross-sectional studies in convenience samples. We will conduct a prospective longitudinal study of the associations between coping behaviors and subsequent anxiety and depressive symptoms during the COVID-19 pandemic in a representative sample of the Spanish general adult population.

**Methods:** We will recruit 1,000 adult participants from all autonomous communities of Spain and with sex, age, and urbanicity distributions similar to those of their populations and assess anxiety and depressive symptoms and coping behaviors using fortnightly questionnaires and real-time methods (ecological momentary assessments) for 1 year. The fortnightly questionnaires will inquire about anxiety and depressive symptoms [General Anxiety Disorder-7 (GAD-7) and Patient Health Questionnaire-9 (PHQ-9)] and the frequency of 10 potential coping behaviors (e.g., follow a routine) during the past 2 weeks. In addition, we will collect several variables that could confound or moderate these associations. These will include subjective well-being [International Positive and Negative Affect Schedule Short Form (I-PANAS-SF) and Satisfaction with Life Scale (SWLS)], obsessive-compulsive symptoms [Obsessive Compulsive Inventory-Revised (OCI-R)], personality and emotional intelligence [International Personality Item Pool (IPIP) and Trait Emotional Intelligence Questionnaire Short Form (TEIQue-SF)], sociodemographic factors (e.g., work status, housing-built environment), and COVID-19 pandemic-related variables (e.g., hospitalizations or limitations in social gatherings). Finally, to analyze the primary relationship between coping behaviors and subsequent anxiety and depressive symptoms, we will use autoregressive moving average (ARMA) models.

**Discussion:** Based on the study results, we will develop evidence-based, clear, and specific recommendations on coping behaviors during the COVID-19 pandemic and lockdown. Such suggestions might eventually help health bodies or individuals to manage current or future pandemics.

## Introduction

Worldwide, the coronavirus disease 2019 (COVID-19) pandemic and subsequent lockdowns might increase anxiety and depressive symptoms. Health bodies provided some recommendations for coping with these symptoms (1). However, most recommendations rely on experiences (e.g., individual prison isolation) that are probably very different from the current situation. The behaviors that may help a prisoner cope with isolation may not aid the general population cope with the pandemic and lockdown. Other recommendations rely on previous research on epidemics, such as the severe acute respiratory syndrome (SARS) epidemic in 2003 or the influenza A pandemic in 2009 (2–5). For instance, Lau and colleagues suggested that positive changes during the SARS epidemic, like spending more time resting, relaxing, or exercising, protected from the negative impacts of the epidemic (3). For other disasters such as terrorist attacks, coping behaviors such as information search and social support have played a role (6). We do not know whether these recommendations apply or could have similar effects in the current context.

Our central hypothesis is that certain “simple” coping behaviors, such as following a healthy diet, or exercise might protect from the adverse psychological effects of the COVID-19 pandemic. Our objective is to find these behaviors, with the final aim to create evidence-based recommendations.

We must note that some research groups have already conducted online surveys to investigate the subject (7). For instance, in April 2020, we conducted a pilot survey of 5,000 Spanish adults 2 weeks after the government established an official lockdown across the country (8). We found that maintaining a healthy/balanced diet and not reading news/updates about COVID-19 very often correlated with lower levels of anxiety and depressive symptoms. Other studies have also found significant associations between certain coping behaviors and fewer mental health problems during the pandemic (9, 10). One study found that anxiety symptoms may mediate the relationships between the threat of COVID-19 and coping behaviors (11). Other studies have reported negative associations between physical activity and general negative emotions, depression, loneliness, or stress (12, 13). And yet others have reported positive associations between social media exposure and anxiety and depression (14).

There are four general limitations in these previous studies. First, most were cross-sectional, i.e., they mainly “correlated” the frequency of behaviors and the severity of symptoms during the same period. Thus, we cannot rule out reverse causation (mistaking a cause for effect and vice versa). For instance, we found that reading news/updates about COVID-19 correlated with increased anxiety. Still, it is uncertain whether individuals first started reading news very often and afterward got anxious, or the other way round, i.e., they got nervous, and after that, they started reading news very often. Second, most studies investigated a short period of the pandemic course. We cannot discard that the relationships might differ depending on whether the pandemic or the lockdown restrictions endure or change. For instance, a longitudinal study in Wuhan residents found that psychological well-being slightly improved after the lockdown was lifted in early April 2020 (15), although the pandemic was not over. Third, most studies inquired about symptoms retrospectively, which might potentially increase recall bias. Finally, most studies used convenience (and not representative) samples.

To overcome these limitations, we will ask participants to answer fortnightly questionnaires for 1 year using a prospective longitudinal design. We aim to assess the association between coping behaviors and subsequent anxiety and depressive symptoms and whether these associations might change with the COVID-19 pandemic and lockdown conditions. Besides, we will also include ecological momentary assessments (EMA) (16), close in time to experience to minimize recall bias. Finally, the sample will be more representative of the Spanish general adult population.

## Methods and Analysis

### Design

We will conduct an observational prospective, longitudinal study in a sample with a demographic distribution like the Spanish general adult population.

### Recruitment

We will recruit a sample of 1,000 individuals who are willing to answer online questionnaires every 2 weeks and EMA for 1 year. The study's target population will be the Spanish general adult population, probably excluding individuals not fluent in Spanish and individuals not reachable by social networks. Inclusion criteria will be: (a) age ≥ 18 years; (b) live in Spain; and (c) have a mobile phone number or email address to receive our notifications. Exclusion criteria will be: (a) fail to answer the preliminary survey or the first online questionnaire; and (b) fail to answer more than eight online questionnaires and/or more than nine EMA during the follow-up.

The sample size estimation relies on the observation that we could detect associations in our pilot study's (8) interim analyses when the sample size was still substantially smaller than 1,000. Thus, while we cannot provide exact sample size/power estimations for the time-series analyses described later, we must note that a sample size of 1,000 would allow the detection of small Pearson correlations (*r* = 0.135) with 90% power after Bonferroni correction for multiple testing (two outcomes × 10 behaviors).

**Figure 1:**
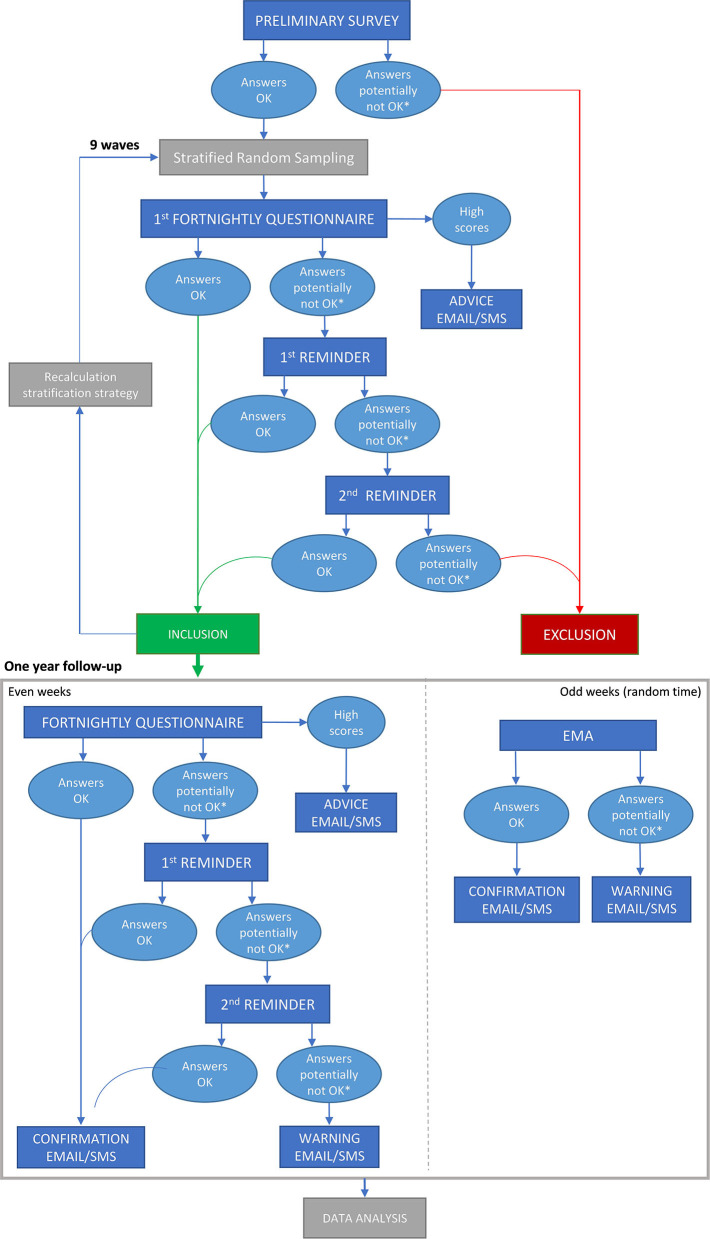
Flowchart of the study. EMA, ecological momentary assessments; SMS, Short Message Service.

Recruitment will follow a two-step strategy (Figure 1). First, we will conduct a “pre-recruitment” step to create a pool of potential participants (who will not have the Spanish population's demographic distribution because some groups will be over-represented). Second, we will conduct a “sampling” step in which, separately for different strata (see next paragraph), we will randomly select several participants so that the final sample has a demographic distribution like the Spanish population.

Similar to previous mental health epidemiological research in the Spanish population (17), stratification factors will include age group (18–34, 35–44, 45–54, 55–64, ≥65-year-old), gender (binary), region (Spanish autonomous community), and urbanicity. We selected these five age groups because each group corresponds to ~7.5 million individuals (18–34 years: 8.6 million; 35–44 years: 7.3 million; 45–54 years: 7.5 million; 55–64 years: 6.3 million; ≥65 years: 9.3 million). For urbanicity, we will use the municipality size threshold (<10,000 residents, vs. higher) used in the UK Household Longitudinal Study, which Pierce et al. used to assess the effects of urbanicity on mental health during the pandemic (18).

#### Pre-recruitment

We will publicize the study on social networks, inviting everyone to participate in a preliminary survey. To reach heterogeneous society sectors, we will ask close and distant acquaintances from varying ages and professions to invite close and distant acquaintances from varying ages and occupations. This preliminary survey will only include the informed consent, the questions about the four stratification variables, the 16 anxiety and depression items [General Anxiety Disorder-7 (GAD-7) (19) and Patient Health Questionnaire-9 (PHQ-9) (11)], and the preferred channel to receive communications [*via* Short Message Service (SMS), email, or both]. We will inform the potential participants that we will randomly select 1,000 individuals and invite them to answer fortnightly questionnaires and EMA for a year, receiving 140€ compensation at the end of the study. We will highlight that only participants included in this second step who complete ≥70% fortnightly questionnaires with <10% missing answers per questionnaire and ≥70% EMAs will receive the compensation.

#### Sampling

The number of participants selected from each specific stratum will depend on the stratum's weight and the overall differences between the sample and the population. We have calculated a “stratum-ideal” number of participants for each stratum that ensures that the sample includes individuals from all strata, but at the same time, larger strata in the population are larger in the sample. However, we will recruit up to five more individuals per stratum than this “stratum-ideal” number if their inclusion reduces the statistically significant differences between the overall sample and the population in age, sex, urbanicity, and region (in this order of priority). To calculate the above “stratum-ideal” number, we retrieved the demographic distribution of the Spanish population from the National Statistics Institute (20) and used the following iterative algorithm: (a) we first set the size of each of the 350 sample strata to one participant; (b) we divided the population strata sizes by the sample strata sizes, obtaining the weight that we would apply to each stratum in a weighted analysis; (c) we added one participant to the stratum with the largest weight; (d) we went back to (b). We will exclude those individuals who do not respond correctly to the first questionnaire.

### Follow-Up

We will contact the selected individuals by SMS and email. On Fridays, Mondays, and following Wednesdays, we will either confirm the participants that we have correctly received their responses, inform them that their responses were incomplete (≥10% missing answers) or potentially inaccurate/inconsistent, or remind them to answer the questionnaire.

We will use two strategies to detect potentially inaccurate/inconsistent responses (i.e., indicators that the participant may have paid low attention). On the one hand, we will look for significant discrepancies between two repeated questions included in each questionnaire (e.g., answering “not at all” and “nearly every day” to the same question). On the other hand, we will detect questionnaires answered in a few seconds, suggesting that the participant did not even read the questions.

Previous research has found that item 9 of PHQ9 (death thoughts) predicts risk for suicide attempts across age groups (21). Therefore, we will send emails/SMS to individuals reporting any frequency on this item advising them to check with their health care provider or, in case of thinking about harming themselves, to go to an emergency service. That said, we will not exclude any individual based on his/her scores because we aim to represent the whole adult population.

### Online Questionnaires and EMA

We will collect all participants' information through an institutional safe online platform (https://enquesta.clinic.cat/). We have created a survey for the “pre-recruitment” step, several questionnaires for the fortnightly follow-up assessments, and a short two-item form for the EMA. Every week, we will send emails and SMS to the participants to answer a questionnaire or an EMA using the platform.

We will not collect any personal information from the participants. Instead, the Uniform Resource Locator (URL) links in the emails and SMS will include a unique random series of letters and numbers to determine which answers correspond to the same participant across the various questionnaires and EMA.

We will present the fortnightly questionnaires every 2 Wednesdays. We understood that Wednesday is neither too close nor too far from the weekend, and thus the answers will not be biased toward weekend or workday memories. The questionnaires will be short to increase the likelihood of response. The first page will include the 16 GAD-/PHQ-9 items, the second page some questions about secondary variables, and the third page the 10 behaviors and the two repeated questions. We will inquire about symptoms first to avoid response order bias (22).

We will prompt the participants to answer the EMA, asked at any random time from Wednesday to Tuesday, from 10 am to 6 pm.

We will make minor modifications to this schedule to avoid special days such as Christmas.

### Main Variables

The study's main dependent variables will be the anxiety and depressive symptoms (GAD-7 and PHQ-9). We will collect them every week: on the first page of the fortnightly questionnaires in even weeks and on the EMA in odd weeks. For the latter, we will only ask one representative item of each scale in the present tense (“Do you feel nervous, anxious, or on edge?,” “Do you feel down, depressed, or hopeless?”).

The independent variables will be the frequency of these 10 behaviors: follow a routine, talk with relatives/friends not living in the household, physical exercise, follow a healthy/balanced diet, drinking water to hydrate, read news/updates about COVID-19 very often, take the opportunity to pursue hobbies or conduct home tasks such as arranging the wardrobe, stay outdoors or look outside, do relaxing activities, and interact with other individuals of the household (e.g., partner or children) (8). We chose these behaviors based on our previous study (8). We declined the possibility that participants could propose other behaviors because we ask all behaviors in all fortnightly questionnaires, but the participants join the study at different times.

### Secondary Variables

In the fortnightly questionnaires, we will also collect several other variables for their use as covariates, modulators, or alternative outcomes.

Assessments of psychological well-being other than anxiety and depression will include the 10-item International Positive and Negative Affect Schedule Short Form (I-PANAS-SF) (23), the 5-item Satisfaction with Life Scale (SWLS) (24), and the 18-item Obsessive Compulsive Inventory-Revised (OCI-R) (25). We will present subjective well-being scales (I-PANAS-SF and SWLS) every 6 weeks and the OCI-R twice (with a 6-month difference between the assessments). In the context of the current anti-pandemic frequent hand-washing recommendations, we removed the two contamination/washing questions (items 11 and 17) from the latter. The reason to include the OCI-R is that pandemic contamination/hygienic measures may increase obsessive-compulsive symptoms.

We will also assess personality and emotional intelligence twice (with a 6-month difference between the assessments) with the 50-item International Personality Item Pool (IPIP) (26) and the Trait Emotional Intelligence Questionnaire Short Form (TEIQue-SF) (27). We assume that personality and emotional intelligence may modulate the individual response and adaption to the crisis. Still, pandemic stress and restrictions could also modulate our personality and emotional intelligence.

To estimate the behaviors' baseline frequency, we will ask the participants to think about their everyday life the two last weeks before the pandemic outbreak.

We will also ask some general factors that may have an influence. We will include questions at the beginning of the study and questions about their change for factors that we considered might vary. They will be the work and telework status, having minor or other dependent people in charge, COVID-19 diagnosis/severity/risk in one-self and close relatives, COVID-19 unrelated life events, and having received mental health treatment for more than 3 months. Conversely, we will include questions only once for factors that we considered more stable. They will consist of years of education, housing-built environment, household age composition, stance on vaccination, and COVID-19-related deaths in close relatives and acquaintances (dates asked at the end of the study). To assess the housing built environment, we will use the items used by Amerio et al. (e.g., housing dimension, livable outdoor space, views, indoor quality) to investigate its effects on mental health during COVID-19 lockdown (28).

Finally, we will collect variables related to the effects of the pandemic and lockdown measures, both at the national level and separately for regions. We will download COVID-19 incidence, percentage of positive tests, the instantaneous reproduction number, hospitalizations, intensive care unit (ICU) admissions, and deaths from the Health Alert and Emergency Coordination Center (29). We will standardize (30) the lockdown restrictions (e.g., limitations in social gatherings) from autonomous communities governments (31–52), and we will estimate the number and emotional valence of Spain-originated tweets with relevant keywords (e.g., “coronavirus” or “vaccine”) (53).

We acknowledge that many other factors may have an influence, e.g., the use of alcohol. However, we chose to limit the number of questions to keep the questionnaires short. Besides, the study's main aim is to link coping behaviors with symptom improvement, and these factors are only secondary variables.

### Data Analysis

The primary analysis will estimate the relationship between coping behaviors and subsequent symptoms. We will use low-order autoregressive moving average (ARMA) models, which attempt to answer how past values and moving averages (e.g., the average of the current and past 2 weeks) explain the present symptoms.

Before the analysis, we will do multiple imputations of the missing responses. In addition, we will also analyze the characteristics of missing data (e.g., the relationship with other variables).

We will account for potential confounds, baseline values, and the effect of potentially modulating factors. To assess whether the results might depend on methodological factors, we will conduct the following sensitivity analyses: (a) using GAD-7 and PHQ-9 from fortnightly questionnaires or the following EMA; (b) weighting or not weighting the strata for their population/sample ratio; (c) excluding the questionnaires or participants with highly potentially inaccurate/inconsistent answers.

For the analyses of alternative outcomes (e.g., I-PANAS-SF), we will have fewer measurements of the outcomes than measurements of coping behaviors. To circumvent this problem, we will time-weighted-average the coping behaviors measured since the last measure of the outcome. Besides, fewer assessments might not allow us to fit ARMA models. In this case, we will fit simpler models (e.g., a pre-post repeated-measure linear model). We also plan a series of other secondary analyses, such as the relationship between the symptoms or restrictions and subsequent personality or emotional intelligence changes.

We will correct the primary analyses for multiple comparisons, and we will conduct all analyses with R (54).

### Creation of Recommendations

The results of this longitudinal study will allow us to create evidence-based recommendations. Specifically, we will grade the main results according to their effect size, statistical significance, and variation in the sensitivity analyses. When the modulator analyses show that the effects might be sensibly different depending on a modulator, we will grade the results separately. Finally, we will write the most robust results as recommendations. We will send them to health organizations and providers, and public policymakers, along with a text explaining the study and the grading.

## Discussion

This project aims to study the relationship between a set of simple potential coping behaviors and subsequent anxiety and depressive symptoms in the context of the COVID-19 pandemic and associated lockdown restrictions. To this end, we will recruit a sample with a demographic distribution like the population and ask the participants to answer fortnightly questionnaires for 1 year and EMA in real-time to estimate the symptoms better.

Based on the study results, we will develop clear and specific recommendations about which coping behaviors may help us mitigate the anxiety and depressive symptoms during the COVID-19 pandemic and lockdown. We will then share these recommendations with health organizations, providers, and public policymakers.

For each of the 20 relationships of interest (i.e., 10 coping behaviors modifying two primary outcomes), the results may draw different scenarios. We will only exemplify the relationship between reading news/updates about COVID-19 and subsequent anxiety symptoms for simplicity. However, we understand that these interpretations would be similar for the other relationships.

A basic scenario could be that we observe that when individuals read news/updates about COVID-19 very often, their anxiety subsequently increases. This finding would support previous interpretations that reading news/updates about COVID-19 increases anxiety (8), and thus the recommendations to decrease the frequency of this behavior (1). However, we could also find that when individuals report high anxiety, they subsequently increase the frequency of reading news/updates about COVID-19. This bidirectional relationship would lead to a vicious circle: anxiety makes individuals read COVID-19 news more often, in turn making them more anxious. Vicious circles are indeed common in anxiety. For instance, anxiety may lead to avoiding feared stimuli, which in turn increases anxiety (55). We should then encourage people to avoid reading news/updates about COVID-19 “too often.”

We may alternatively observe that when individuals read news/updates about COVID-19 very often, their anxiety subsequently decreases. This finding would question previous studies, up to the point that we should wonder whether we should recommend reading COVID-19 news often to prevent anxiety. In additional analyses, we could also find that when individuals report high anxiety, they subsequently increase the frequency of reading COVID-19 news, thus suggesting reverse causation. We could speculate that individuals with COVID-19-related anxiety learn that their anxiety decreases after reading COVID-19 news. This amelioration would be the reason why they read COVID-19 news more often.

We may also find that the effect of reading “too often” COVID-19 news is modulated by personality traits, which we also investigate in our study. This result would not be surprising because each Big Five personality is associated with different coping strategies. For example, extraversion and conscientiousness are related to problem-solving and cognitive restructuring. In contrast, neuroticism is related to wishful thinking, withdrawal, and emotion-focused coping (56). Therefore, we could offer more personalized suggestions (i.e., considering coping strategies and personality traits). Another potential modulator of the relationships between coping behaviors and subsequent anxiety or depressive symptoms may be emotional intelligence. A recent study in the context of the COVID-19 pandemic showed indeed that emotional intelligence is related to all coping strategies (57).

GAD-7 and PHQ9-9 ask about anxiety and depressive symptoms during the previous 2 weeks. Therefore, we expect that the GAD-7 and PHQ9-9 scores will strongly correlate with the anxiety and depression levels measured with the EMA collected during those 2 weeks. However, we cannot give this for granted because there may be some recall bias. For example, a recent study in patients with major depressive disorder found that the severity of suicidal ideation assessed through 1-week EMA correlated with the ideation scores retrospective collected at the end of the week. Still, many participants reporting ideation with EMA denied ideation on the retrospective assessment (58). Thus, the analysis of the relationship between behaviors and EMA scores will yield light on the relevance of this potential recall bias. Suppose the results are similar to those obtained using GAD-7 and PHQ9-9. In that case, we will conclude that the potential recall bias has no significant effects on our analyses. Conversely, if the results are substantially different, we will report and interpret both results differently.

We believe that the secondary analyses may also provide interesting guidance. For instance, we may find that some coping behaviors have an only weak relationship with subsequent anxiety symptoms but a strong connection with obsessive-compulsive symptoms. We chose this outcome for its relevance during the pandemic; some studies have indeed observed an increase in obsessive-compulsive symptoms (59). Other interesting alternative outcomes will be subjective well-being, personality, and emotional intelligence. Personality changes little during adulthood (60), but a few studies have found that catastrophic trauma may potentially lead to personality changes (61).

This project has some potential limitations. First, we may face difficulties in recruiting the target sample. The recruitment may seem straightforward if one keeps in mind that we will recruit 1,000 participants in several weeks, while in the pilot study, we recruited 5,000 participants in <2 weeks. However, in the pilot study, we only presented a single survey to a convenience sample. Here, we will conduct a longitudinal study in a sample with a demographic distribution like the population. Second, as in any voluntary study, more altruistic individuals may tend to participate more, although the compensation should reduce this potential participation bias. Third, a common problem of longitudinal studies is the loss of follow-up. To minimize this risk, we have created short questionnaires, and we will only compensate economically the participants who complete the follow-up. Fourth, we acknowledge the possibility that participants modify their behavior in response to the assessments (i.e., the Hawthorne effect) (62). However, we will not focus on the behavioral changes but on the impact of these changes on anxiety and depressive symptoms. Finally, we must acknowledge that while this study will assess whether coping behaviors precede changes in symptoms, it is only observational. We would need a randomized controlled design to evaluate the “true” efficacy of coping behaviors in reducing anxiety and depressive symptoms.

## Ethics Statement

The study was reviewed and approved by Comitè d'Ètica de la Investigació amb medicaments, Hospital Clínic de Barcelona (protocol HCB/2020/0890). The participants will provide their electronic informed consent to participate in this study.

## Author Contributions

JR, MF, LF, AS, JA, CT, CB, DH-M, IG, and EV conceived the study. All authors participated in the redaction of the manuscript including minor or major modifications of the protocol.

## Conflict of Interest

IG has received grants and served as consultant, advisor, or CME speaker for the following identities: Angelini, AstraZeneca, CasenRecordati, Ferrer, Janssen Cilag, and Lundbeck, Lundbeck-Otsuka, SEI Healthcare, FEDER. EV has received grants and served as consultant, advisor, or CME speaker for the following entities (work unrelated to the topic of this manuscript): AB-Biotics, Abbott, Allergan, Angelini, Dainippon Sumitomo Pharma, Galenica, Janssen, Lundbeck, Novartis, Otsuka, Sage, Sanofi-Aventis, and Takeda. The remaining authors declare that the research was conducted in the absence of any commercial or financial relationships that could be construed as a potential conflict of interest.

## References

[1] CDC. Coronavirus Disease 2019 (COVID-19) - Stress and Coping (2020). Available online at: https://www.cdc.gov/coronavirus/2019-ncov/daily-life-coping/managing-stress-anxiety.html

[2] Lee TM Chi I Chung LW Chou KL. Ageing and psychological response during the post-SARS period. Aging Ment Health. (2006) 10:303–11. 10.1080/13607860600638545 16777659

[3] Lau JT Yang X Tsui HY Pang E Wing YK. Positive mental health-related impacts of the SARS epidemic on the general public in Hong Kong and their associations with other negative impacts. J Infect. (2006) 53:114–24. 10.1016/j.jinf.2005.10.019 16343636 PMC7132442

[4] Matsuishi K Kawazoe A Imai H Ito A Mouri K Kitamura N. Psychological impact of the pandemic (H1N1) 2009 on general hospital workers in Kobe. Psychiatry Clin Neurosci. (2012) 66:353–60. 10.1111/j.1440-1819.2012.02336.x 22624741

[5] Taha S Matheson K Cronin T Anisman H. Intolerance of uncertainty, appraisals, coping, and anxiety: the case of the 2009 H1N1 pandemic. Br J Health Psychol. (2014) 19:592–605. 10.1111/bjhp.12058 23834735

[6] Bleich A Gelkopf M Solomon Z. Exposure to terrorism, stress-related mental health symptoms, and coping behaviors among a nationally representative sample in Israel. JAMA. (2003) 290:612–20. 10.1001/jama.290.5.612 12902364

[7] Holmes EA O'Connor RC Perry VH Tracey I Wessely S Arseneault L. Multidisciplinary research priorities for the COVID-19 pandemic: a call for action for mental health science. Lancet Psychiatry. (2020) 7:547–60. 10.1016/S2215-0366(20)30168-1 32304649 PMC7159850

[8] Fullana MA Hidalgo-Mazzei D Vieta E Radua J. Coping behaviors associated with decreased anxiety and depressive symptoms during the COVID-19 pandemic and lockdown. J Affect Disord. (2020) 275:80–1. 10.1016/j.jad.2020.06.027 32658829 PMC7329680

[9] Solé B Verdolini N Amoretti S Montejo L Rosa AR Hogg B. Effects of the COVID-19 pandemic and lockdown in Spain: comparison between community controls and patients with a psychiatric disorder. Preliminary results from the BRIS-MHC STUDY. J Affect Disord. (2020) 11:99. 10.1016/j.jad.2020.11.099 33279864 PMC7683299

[10] Guo J Feng XL Wang XH Ivan MH Coping with COVID-19: exposure to COVID-19 and negative impact on livelihood predict elevated mental health problems in Chinese adults. Int J Environ Res Public Health. (2020) 17:113857. 10.3390/ijerph17113857 PMC7312167 32485859

[11] Cypryanska M Nezlek JB. Anxiety as a mediator of relationships between perceptions of the threat of COVID-19 and coping behaviors during the onset of the pandemic in Poland. PLoS ONE. (2020) 15:e0241464. 10.1371/journal.pone.0241464 33125435 PMC7599044

[12] Zhang Y Zhang H Ma X Di Q. Mental health problems during the COVID-19 pandemics and the mitigation effects of exercise: a longitudinal study of college students in China. Int J Environ Res Public Health. (2020) 17:3722. 10.3390/ijerph17103722 32466163 PMC7277113

[13] Meyer J McDowell C Lansing J Brower C Smith L Tully M. Changes in physical activity and sedentary behavior in response to COVID-19 and their associations with mental health in 3,052 US adults. Int J Environ Res Public Health. (2020) 17:186469. 10.3390/ijerph17186469 PMC7559240 32899495

[14] Gao J Zheng P Jia Y Chen H Mao Y Chen S. Mental health problems and social media exposure during COVID-19 outbreak. PLoS ONE. (2020) 15:e0231924. 10.1371/journal.pone.0231924 32298385 PMC7162477

[15] Zhou T Nguyen TT Zhong J Liu J. A COVID-19 descriptive study of life after lockdown in Wuhan, China. Royal Soc Open Sci. (2020) 7:200705. 10.1098/rsos.200705 33047032 PMC7540789

[16] Ludvigsson JF. Case report and systematic review suggest that children may experience similar long-term effects to adults after clinical COVID-19. Acta Paediatr. (2020) 2020:15673. 10.1111/apa.15673 33205450 PMC7753397

[17] Haro JM Palacín C Vilagut G Martínez M Bernal M Luque I. Prevalence of mental disorders and associated factors: results from the ESEMeD-Spain study. Med Clin. (2006) 126:445–51. 10.1157/13086324 16620730

[18] Pierce M Hope H Ford T Hatch S Hotopf M John A. Mental health before and during the COVID-19 pandemic: a longitudinal probability sample survey of the UK population. Lancet Psychiatry. (2020) 7:883–92. 10.1016/S2215-0366(20)30308-4 32707037 PMC7373389

[19] Spitzer RL Kroenke K Williams JB Lowe B. A brief measure for assessing generalized anxiety disorder: the GAD-7. Arch Intern Med. (2006) 166:1092–7. 10.1001/archinte.166.10.1092 16717171

[20] Instituto Nacional de Estadística. Población Residente por Fecha, Sexo y Edad. (2020) Available online at: https://www.ine.es/jaxiT3/Tabla.htm?t=31304

[21] Rossom RC Coleman KJ Ahmedani BK Beck A Johnson E Oliver M. Suicidal ideation reported on the PHQ9 and risk of suicidal behavior across age groups. J Affect Disord. (2017) 215:77–84. 10.1016/j.jad.2017.03.037 28319695 PMC5412508

[22] Israel GD Taylor CL. Can response order bias evaluations? Eval Progr Plan. (1990) 13:365–71. 10.1016/0149-7189(90)90021-N

[23] Thompson ER. Development and validation of an internationally reliable short-form of the positive and negative affect schedule (PANAS). J Cross-Cult Psychol. (2007) 38:227–42. 10.1177/0022022106297301

[24] Diener E Emmons RA Larsen RJ Griffin S. The satisfaction with life scale. J Pers Assess. (1985) 49:71–5. 10.1207/s15327752jpa4901_13 16367493

[25] Foa EB Huppert JD Leiberg S Langner R Kichic R Hajcak G. The obsessive-compulsive inventory: development and validation of a short version. Psychol Assess. (2002) 14:485–96. 10.1037/1040-3590.14.4.485 12501574

[26] De Oliveira R Cherubini M Oliver N. Influence of personality on satisfaction with mobile phone services. ACM Trans Comp Hum Interact. (2013) 10:2463581. 10.1145/2463579.2463581

[27] Laborde S Allen MS Guillén F Construct and concurrent validity of the short- and long-form versions of the trait emotional intelligence questionnaire. Personal Individ Differ. (2016) 101:232–5. 10.1016/j.paid.2016.06.009

[28] Amerio A Brambilla A Morganti A Aguglia A Bianchi D Santi F. COVID-19 lockdown: housing built environment's effects on mental health. Int J Environ Res Public Health. (2020) 17:165973. 10.3390/ijerph17165973 32824594 PMC7459481

[29] Centro de Coordinación de Alertas y Emergencias Sanitarias. Situación Actual (2020). Available online at: https://www.mscbs.gob.es/profesionales/saludPublica/ccayes/alertasActual/nCov/situacionActual.htm

[30] Bolton MJ Chapman BP Van Marwijk H. Low-dose naltrexone as a treatment for chronic fatigue syndrome. BMJ Case Rep. (2020) 13:232502. 10.1136/bcr-2019-232502 31911410 PMC6954765

[31] Junta de Andalucía. Normativa COVID-19 Andalucía (2020). Available online at: https://www.juntadeandalucia.es/servicios/normativa/covid-19.html

[32] Gobierno de Aragón. Normativa Coronavirus COVID-19 (2020). Available online at: https://www.aragon.es/coronavirus/normativa

[33] Gobierno de Aragón. Alerta sanitaria derivada de la COVID-19 en Aragón (2020). Available online at: https://www.aragon.es/-/nueva-normalidad-derivada-de-la-covid-19-en-aragon

[34] Gobierno del Principado de Asturias. Disposiciones BOPA en materia de coronavirus COVID-19 (2020). Available online at: https://coronavirus.asturias.es/bopa1

[35] Govern de les Illes Balears. Mesures Especials Mallorca, Menorca, Formentera, Eivissa (2020). Available online at: https://www.caib.es/sites/covid-19/ca/actualitzacion_de_medidas/

[36] Gobierno de Canarias. Materiales informativos - Nueva Normalidad (2020). Available online at: https://www3.gobiernodecanarias.org/sanidad/scs/contenidoGenerico.jsp?idDocument=f105952f-b466-11ea-8548-7f4ff485ed65&idCarpeta=e01092c2-7d66-11ea-871d-cb574c2473a4

[37] Gobierno de Cantabria. Recopilación de anuncios del Gobierno de Cantabria relacionados con la pandemia por COVID-19 (2020). Available online at: https://www.scsalud.es/documents/2162705/9234715/Recopilaci%C3%B3n+de+anuncios+BOC+COVID-19.pdf/63d855da-1fb3-0372-6b1a-5da2a5121e7e?t=1606289688685

[38] Junta de Castilla y León. Infografías y normativa COVID-19 (2020). Available online at: https://www.jcyl.es/web/es/portada/informacion-coronavirus/infografias-normativa-covid19.html

[39] Junta de Comunidades de Castilla-La Mancha. Normativa sobre crisis sanitaria COVID-19 (2020). Available online at: https://docm.castillalamancha.es/portaldocm/listadoCOVID.do

[40] Junta de Comunidades de Castilla-La Mancha. Medidas especiales de contención frente a COVID-19 (2020). Available online at: https://sanidad.castillalamancha.es/ciudadanos/enfermedades-infecciosas/coronavirus/transicion-hacia-una-nueva-normalidad/medidas-especiales-de-contencion-frente-covid-19

[41] Generalitat de Catalunya. Mesures i restriccions COVID-19 (2020). Available online at: https://web.gencat.cat/ca/activem/restriccions-territorials/catalunya/index.html

[42] Generalitat de Catalunya. Normativa sobre la situació de pandèmia pel SARS-CoV-2 (2020). Available online at: https://portaljuridic.gencat.cat/ca/pjur_ocults/pjur_CoV-2/

[43] Generalitat Valenciana. Info coronavirus (2020). Available online at: http://infocoronavirus.gva.es/va/infocoronavirus

[44] Región de Murcia. Regulación normativa COVID Región de Murcia (2020). Available online at: https://www.murciasalud.es/recursos/ficheros/468559-20201125_Regulacion_normativa_COVID_RM.pdf

[45] Región de Murcia. COVID-19: legislación BORM (2020). Available online at: https://www.murciasalud.es/seccion.php?mod=secciones&op=ver_mes&anio=2020&mes=10&idsec=6534

[46] Junta de Extremadura. Información sobre COVID-19. (2020). Available online at: https://ciudadano.gobex.es/

[47] Junta de Extremadura. Información sobre las medidas de gestión de la crisis sanitaria COVID-19 (2020). Available online at: https://ciudadano.gobex.es/noticias/-/noticia/ficha/9792520

[48] Xunta de Galicia. Restriccións COVID-19 (2020). Available online at: https://coronavirus.sergas.es/Contidos/Xerais

[49] Comunidad de Madrid. 2019-Nuevo Coronavirus (2020). Available online at: https://www.comunidad.madrid/servicios/salud/2019-nuevo-coronavirus

[50] Gobierno de la Rioja. Información práctica para ciudadanos y empresas durante el coronavirus (2020). Available online at: https://actualidad.larioja.org/coronavirus

[51] Ciudad Autónoma de Ceuta. Nuevas Restricciones (2020). Available online at: https://www.ceuta.es/ceuta/nuevas-restriccciones

[52] Ciudad Autónoma de Melilla. Medidas adoptadas por la Ciudad Autónoma de Melilla (2020). Available online at: https://www.melilla.es/melillaPortal/contenedor.jsp?seccion=s_ldoc_d1_v1.jsp&codbusqueda=776&language=es&codResi=1&codMenuPN=601&codMenuSN=730&codMenu=739&layout=contenedor.jsp

[53] Chew C Eysenbach G. Pandemics in the age of Twitter: content analysis of Tweets during the 2009 H1N1 outbreak. PLoS ONE. (2010) 5:e14118. 10.1371/journal.pone.0014118 21124761 PMC2993925

[54] R Core Team. R: A Language and Environment for Statistical Computing. Vienna: R Foundation for Statistical Computing (2020).

[55] Krypotos AM Effting M Kindt M Beckers T. Avoidance learning: a review of theoretical models and recent developments. Front Behav Neurosci. (2015) 9:189. 10.3389/fnbeh.2015.00189 26257618 PMC4508580

[56] Connor-Smith JK Flachsbart C. Relations between personality and coping: a meta-analysis. J Pers Soc Psychol. (2007) 93:1080–107. 10.1037/0022-3514.93.6.1080 18072856

[57] Prentice C Zeidan S Wang X. Personality trait EI and coping with COVID 19 measures. Int J Disast Risk Reduct. (2020) 51:101789. 10.1016/j.ijdrr.2020.101789 32834975 PMC7418749

[58] Gratch I Choo TH Galfalvy H Keilp JG Itzhaky L Mann JJ. Detecting suicidal thoughts: the power of ecological momentary assessment. Depress Anxiety. (2021) 38:8–16. 10.1002/da.23043 32442349

[59] Cox RC Olatunji BO. Linking insomnia and OCD symptoms during the coronavirus pandemic: examination of prospective associations. J Anxiety Disord. (2021) 77:102341. 10.1016/j.janxdis.2020.102341 33285369 PMC7689352

[60] Ferguson CJ. A meta-analysis of normal and disordered personality across the life span. J Pers Soc Psychol. (2010) 98:659–67. 10.1037/a0018770 20307136

[61] Munjiza J Law V Crawford MJ. Lasting personality pathology following exposure to catastrophic trauma in adults: systematic review. Personal Mental Health. (2014) 8:320–36. 10.1002/pmh.1271 25123294

[62] McCambridge J Witton J Elbourne DR. Systematic review of the Hawthorne effect: new concepts are needed to study research participation effects. J Clin Epidemiol. (2014) 67:267–77. 10.1016/j.jclinepi.2013.08.015 24275499 PMC3969247

